# Nutrigenetic Contributions to Dyslipidemia: A Focus on Physiologically Relevant Pathways of Lipid and Lipoprotein Metabolism

**DOI:** 10.3390/nu10101404

**Published:** 2018-10-02

**Authors:** Bridget A. Hannon, Naiman A. Khan, Margarita Teran-Garcia

**Affiliations:** 1 Division of Nutritional Sciences, University of Illinois at Urbana-Champaign, Urbana-Champaign, IL 61801, USA; bhannon3@illinois.edu (B.A.H.); nakhan2@illinois.edu (N.A.K.); 2 Department of Kinesiology and Community Health, University of Illinois at Urbana-Champaign, Urbana-Champaign, IL 61801, USA; 3 Department of Human Development and Family Studies, Cooperative Extension, University of Illinois at Urbana-Champaign, Carle Illinois College of Medicine, Urbana-Champaign, IL 61801, USA

**Keywords:** dyslipidemia, nutrigenetics, lipids

## Abstract

Cardiovascular disease (CVD) remains the number one cause of death worldwide, and dyslipidemia is a major predictor of CVD mortality. Elevated lipid concentrations are the result of multiple genetic and environmental factors. Over 150 genetic loci have been associated with blood lipid levels. However, not all variants are present in pathways relevant to the pathophysiology of dyslipidemia. The study of these physiologically relevant variants can provide mechanistic understanding of dyslipidemia and identify potential novel therapeutic targets. Additionally, dietary fatty acids have been evidenced to exert both positive and negative effects on lipid profiles. The metabolism of both dietary and endogenously synthesized lipids can be affected by individual genetic variation to produce elevated lipid concentrations. This review will explore the genetic, dietary, and nutrigenetic contributions to dyslipidemia.

## 1. Introduction

Elevated blood lipid concentrations, or dyslipidemia, currently affect 13% of the US population, and are strong predictors of cardiovascular disease (CVD) [1]. Dyslipidemia can be diagnosed by the presence of one or more of the following phenotypes: elevated concentrations of total cholesterol (TC), low-density lipoprotein cholesterol (LDL), and triglycerides (TG), or low concentrations of high-density lipoprotein cholesterol (HDL) [2]. Dyslipidemia is a complex disease that is the result of multiple biological and behavioral etiologies, such as genetic predisposition, metabolic capacity, dietary intake, and physical activity [3]. Understanding the interactions between these complex factors to produce phenotypes of dyslipidemia is crucial to identifying and implementing successful strategies to manage blood lipids. Among biological factors, the study of genetics is essential to improving scientific understanding of disease progression at its most basic level and understanding of the role of individual genetic variation in disease predisposition can lead to improvements in identification and prevention of disease in genetically at-risk individuals. Of the many behavioral contributors to dyslipidemia, diet offers one of the most efficacious behavioral approaches to disease prevention, and it is a crucial determinant of maintenance of health throughout the lifespan [4]. The role of dietary fat intake in exacerbation or amelioration of CVD risk has been a topic of debate in the field of nutrition. Dietary fatty acids are a heterogeneous group of nutrients, and their varying molecular properties, as well as the food matrix in which they reside, exert differential effects on blood lipids. The intake and metabolism of various fatty acids may be influenced by individual genetic variation. These biological and behavioral factors must be considered not as individual risk factors, but as interacting elements. The following review of the literature will present a summary of the recent work conducted to better elucidate the role of both biological (genetic) and behavioral (dietary) influences on dyslipidemia, and the interactions of these components in the clinical intervention setting.

## 2. Pathophysiology of Dyslipidemia 

Elevated TG and decreased HDL concentrations are metabolic consequences of excess visceral adipose tissue and increase risk of atherosclerotic disease through various mechanisms. The role of elevated TG concentrations in CVD progression is not fully elucidated, but it has been postulated to be due to increased endothelial activation and inflammation [5]. Elevated TG concentrations are strongly associated with insulin resistance, CVD, and other indicators of metabolic dysfunction, due to excess adipose tissue mass [6,7]. A hypertriglyceridemic state promotes the exchange of TG from very-low density lipoprotein (VLDL) for cholesterol esters from LDL and HDL particles, creating small, lipid-poor particles. Small HDL particles are more susceptible to degradation, thus contributing to the low HDL concentrations observed in the presence of other dyslipidemias [8]. Elevated HDL concentrations are generally recognized as cardioprotective, as these lipoproteins serve to sequester excess cholesterol to the liver for excretion. Low concentrations of HDL are a diagnostic biomarker for both the Metabolic Syndrome (MetS) and CVD. The relationship between elevated HDL and metabolic disease has been challenged by results from clinical trials with HDL-raising agents, which did not lead to reduction in cardiovascular events compared to the control group [9]. However, due to the strong inverse relationship between HDL concentrations and CVD at the epidemiological level, it remains a key biomarker for assessing cardiometabolic health [10].

## 3. Genetic Contributions to Dyslipidemia

**Figure 1:**
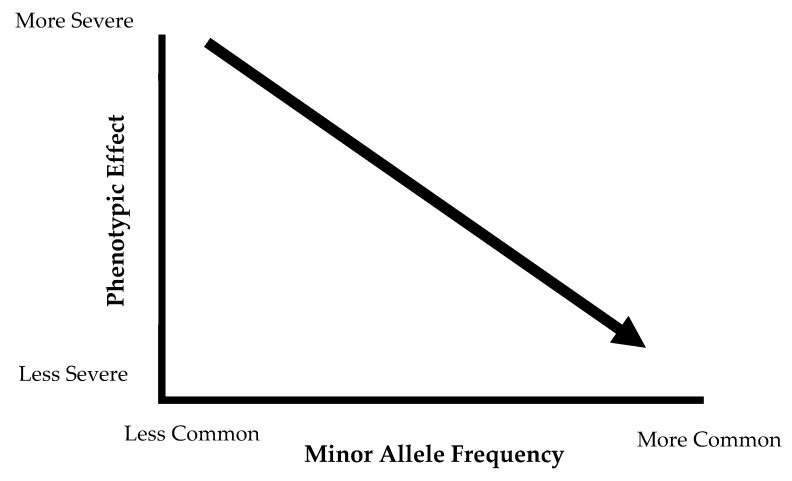
Graphic representation of phenotypic effects of rare versus common variants. Rare variants, such as monogenic disorders, fall on the left of the graph. Common variants with a less severe phenotypic effects are on the right.

With the exception of rare genetic mutations, the majority of dyslipidemias are secondary to other metabolic abnormalities, including abdominal obesity [6]. When describing the complex genetic components of dyslipidemia, one can distinguish between monogenic and polygenic traits. Monogenic diseases are the result of a single mutation in one gene, resulting in a severe phenotype. Some classic examples of these monogenic conditions include Tangier disease (resulting in severely low HDL), LDL receptor deficiency (characterized by elevated LDL concentrations), familial chylomicronemia or lipoprotein lipase (LPL) deficiency (causes severe hypertriglyceridemia) and other familial hypercholesterolemias [11,12,13]. Table 1 presents summary of selected monogenic lipid disorders. These monogenic conditions produce a severe effect, but the frequency of these risk alleles in the population is considerably low. The common variants that produce smaller phenotypic effects contribute to the polygenic nature of obesity and dyslipidemia. These common variants, specifically the single nucleotide polymorphisms (SNPs), are present in at least 1% of the population, and the phenotypic effect of these SNPs individually is not likely to be observed. The differences in phenotypic effect and allele frequency between rare and common variances are represented in Figure 1. Monogenic conditions are represented on the left side of the graph, where the phenotypic effect is very severe, but the frequency is low. The common variants fall on the middle and right side of the graph, with variants exerting a small phenotypic effect that is not deleterious and are present in high frequency in the population. On their own, these variants cannot result in a pronounced phenotype, but the co-occurrence of many of these common variants may cumulatively increase genetic risk for these diseases. Dyslipidemia and atherosclerosis are complex phenotypes, and thus the genetic component of these disease is also the result of complex interactions between various metabolic pathways [14].

These polygenic, common variants associated with dyslipidemia have been identified through genome-wide association studies (GWAS). Over 150 loci have been specifically associated with blood lipid concentrations (total cholesterol (TC), TG, HDL, and LDL) in European populations [16,17]. Notably, several of the identified variants were in biologically and clinically relevant genes, such as angiopoietin-like proteins 3 and 4 (*ANGPTL3/4*), inhibitors of LPL, and *HMGCR*, which codes for 3-hydroxy-3-methylglutaryl-CoA reductase, a target for statin therapy and the rate-limiting enzyme in cholesterol synthesis. GWAS are a powerful and hypothesis-generating tool that can identify loci that are associated with phenotypes of dyslipidemia, research into the effects of the physiological relevance and implication of functional variants in dyslipidemia phenotypes will further increase the understanding of this complex disease.

### 3.1. Focus on Physiological Relevance

**Figure 2:**
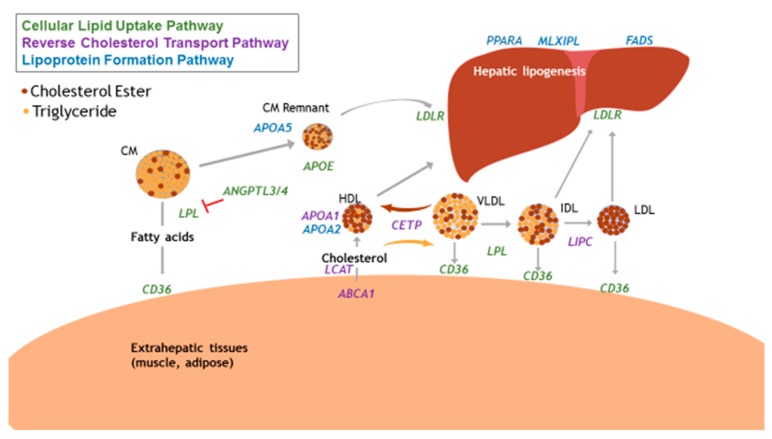
Physiologically relevant genes of lipid and lipoprotein metabolism pathways. CM, chylomicron; HDL, high-density lipoprotein; IDL, intermediate density lipoprotein; LDL, low-density lipoprotein; VLDL, very-low density lipoprotein; *ABCA1*, ATP-binding cassette transported subfamily A member 1; *ANGPTL3/4*, angiopoietin-like proteins 3 & 4; *APOA1,* apolipoprotein AI; *APOA2,* apolipoprotein AII; *APOAV,* apolipoprotein AV; *APOE*, apolipoprotein E; *CD36*, cluster of differentiation 36 (fatty acid translocase); *CETP*, cholesterol esterase transfer protein; *FADS*, fatty acid desaturase cluster; *MLXIPL*, MLX interacting protein like; *LCAT,* lecithin, cholesterol acyltransferase; *LDLR*, LDL receptor; *LIPC*, hepatic lipase; *LPL*, lipoprotein lipase; *PPARA,* peroxisome-proliferator activator receptor alpha.

Not all associated common variants are in physiologically relevant pathways, and therefore cannot provide insight into the mechanisms by which nutrients interact with metabolic processes to produce phenotypes of dyslipidemia. In the pathophysiology and progression of atherogenic dyslipidemia, relevant pathways can include reverse cholesterol transport, cellular lipid uptake, and lipoprotein formation. The interactions of these pathways are depicted in Figure 2.

Reverse cholesterol transport (RCT) facilitates the return of excess cholesterol from peripheral tissues to the liver to be excreted from the body as bile [18]. Key proteins in this pathway include ATP-binding cassette subfamily A member 1 (ABCA1), cholesterol-ester transfer protein (CETP), apolipoprotein A1 (APOA1), hepatic lipase (HL, gene name: *LIPC*), and lecithin: cholesterol acyltransferase (LCAT), which serve to regulate concentrations of HDL and TG in circulation. Altered functionality of the RCT pathway can lead to decreased HDL concentrations, as fewer cholesteryl ester particles are accumulated within HDL particles [19]. SNPs in these genes have been previously associated with blood lipids in various populations. ABCA1 is essential in the efflux of cholesterol from peripheral tissues, and complete knockout of this protein results in Tangier disease. However, this gene contains several common polymorphisms that have been associated with HDL [20] and TG concentrations [21]. Mirmiran et al. recently described the gene-diet interactions of five CETP variants in observational and intervention studies [22]. These authors reported significant interactions between CETP genotype and dietary components, including alcohol and fat intake, to associate with blood lipid profiles. Interestingly, Nakamura et al. has reported evidence for the combined effects of multiple SNPs in the *ABCA1* and *CETP* genes, suggesting a more significant genetic contribution to blood lipid concentrations when these variants are considered together, rather than on their own [23]. APOA1 is the predominant apolipoprotein on HDL particles and essential in RCT function and HDL formation. Variants in this gene have been associated with blood lipids in both European and Chinese populations [16,24]. HL is involved in the remodeling of HDL particles, and thus facilitates RCT [25]. Polymorphisms in the coding and promotor regions of *LIPC* have been identified through GWAS, and subsequently studied for associations with blood lipids in diverse populations [26,27]. SNPs in *LIPC* have also been implicated in affecting lipid response to weight loss interventions [28]. The effect of *LIPC* polymorphisms on blood lipids is more well-defined compared to other genes in this pathway, due to the extensive body of evidence conducted on this gene. LCAT is another protein involved in HDL maturation, as it is responsible for synthesis of cholesteryl ester in plasma. Due to its functional role in RCT, it is logical that the majority of candidate gene studies have focused on HDL as the phenotypic outcome of interest. Significant associations have been detected between *LCAT* polymorphisms and HDL in clinical populations, but there is not substantial evidence to definitively conclude that variants in this gene strongly impact blood lipid concentrations [29]. 

Cellular lipid uptake refers to the movement of dietary or endogenously produced lipids and lipoproteins through circulation, peripheral tissues, and the liver [30,31]. Key proteins in this pathway include lipoprotein lipase (LPL), LDLR, ANGPTL3/4, and fatty acid translocase (cluster of differentiation 36, CD36). LPL is present on cellular membranes and is involved in the lipolysis of TG in lipoproteins to fatty acids. Several common variants in the *LPL* gene have been associated with blood lipids, including a gain-of-function mutation that is associated with TG concentrations in European, but not African, populations [32]. Several SNPs have also been associated with HDL concentrations and high-fat diet [33], indicating the importance of this protein in the metabolism of dietary and endogenous lipids. LDLR is expressed primarily in hepatocytes, and polymorphisms in this gene can affect protein functionality, splicing, or transcription. Associations between variants in *LDLR* and adverse blood lipid concentrations have been detected in GWAS [34], and one variant, rs688, has been studied in vitro to determine the mechanistic consequences of this polymorphism on altered protein functionality [35]. ANGPTL 3 and 4 inhibit LPL in cardiac and skeletal muscle and adipose tissue, preventing the lipolysis and removal of TG from circulation. *ANGPTL3* expression also results in lower LDL production through increased clearance of ApoB-containing lipoproteins [36]. *ANGPTL4* is induced in the fasting state, allowing for increased delivery of fatty acids to tissues other than adipose. Genetic associations between variants in *ANGPTL4* and both LDL [16] and HDL concentrations [37] have been reported in European populations. The consequences of *ANGPTL* variants on dyslipidemia was recently summarized by Paththinige et al. [38]. CD36 is involved in the cellular uptake of both dietary and endogenous lipids, and variants in the *CD36* gene were first associated with blood lipids by Ma et al. [39]. Mechanistically, *CD36* is a logical target for gene-diet interaction studies, and polymorphisms have been associated with blood lipids in diverse populations with [40] and without the inclusion of dietary intake [41].

The endogenous synthesis and export of lipids and lipoproteins from the liver also has clinical relevance in dyslipidemia and obesity, as excess energy intake can upregulate these processes [42,43]. Common variants in these pathways can alter the functionality of encoded proteins, resulting in metabolic alterations and phenotypic traits such as dyslipidemia [44]. Genetic variants present in genes coding for apolipoproteins also have been evidenced to impact risk of dyslipidemia and atherosclerotic disease. The most classic example is *APOE,* coding for apolipoprotein E (APOE)*.* APOE circulates on lipoproteins in both systemic circulation and the central nervous system. The isoforms of this gene affect the affinity of APOE to its binding protein, and the E4 genotype has been associated with increased CVD risk and elevated blood lipid levels, and has been summarized previously [45]. Genes coding for other apolipoproteins also contain common variants previously been associated with blood lipids and have functional relevance for dyslipidemia, such as *APOA5* and *APOA2.* Variants in *APOA5* have been evidenced to significantly impact TG concentrations and were recently summarized by Guardiola and Ribalta [46]. *APOA2* is also associated with HDL, and variants in this gene have been evidenced to interact with dietary fat intake to affect inflammatory status among individuals with diabetes, although the mechanism remains to be elucidated [47,48]. Regarding endogenous lipogenesis, key proteins include fatty acid desaturase (FADS) and peroxisome-proliferator activator receptor alpha (PPARA). Genes in the *FADS* cluster (*FADS1, FADS2*, *FADS3*) code for proteins responsible for desaturation of dietary and endogenous lipids, and variants in these genes have been associated with circulating polyunsaturated fatty acid (PUFA) concentrations, as it is postulated that presence of certain polymorphisms results in decreased functionality of the enzymes [49,50,51]. Additionally, we and others have published on the associations between *FADS* SNPs and blood lipids [52,53]. PPARA regulates a host of lipid and glucose homeostatic processes in the liver, and as PUFAs are ligands for all PPAR isoforms, these genes are targets for gene-diet interaction studies [54]. Variants in *PPARA* have been evidenced to influence blood lipid concentrations in the context of a high-fat diet [55,56]. Regarding nutritional control of hepatic TG synthesis, max-like protein X (MLX) interacting protein like (MLXIPL) induces these pathways in a carbohydrate-dependent manner (an alias for *MLXIPL* is carbohydrate-response element binding protein, ChREBP). Variants in *MLXIPL* have been examined as a mechanism for elevated TG concentrations, and associations have been detected in a Chinese population [57]. However, there are other transcriptional regulators of lipid synthesis, such as sterol-regulatory element binding protein 1 (SREBP1) and upstream transcription factor (USF), that have not been extensively explored in genetic associations. The study of pathways involved in blood lipid concentrations is necessary to better understand of the biological aspects of dyslipidemia and potentially identify new targets in specific proteins or pathways to develop preventative and treatment therapies.

### 3.2. Differences in Minor Allele Frequency and Special Populations

The majority of genetic association studies have been conducted among individuals of European descent; as of 2011, only 4% of GWAS had been conducted in non-European populations [58]. Evidence from genetic association studies in non-European populations have concluded that findings from one study may not always apply to other populations. Examples of this include differences in minor allele frequency (MAF) across populations, differences in risk allele, and discovery of novel candidate loci. The study of genetic associations with dyslipidemia is necessary to better understand the biological reasons for increased disease prevalence among certain ethnic groups. The Mexican population has one of the highest prevalence of dyslipidemias, with low HDL and elevated TG concentrations affecting 61% and 32% of the adult population, respectively [59]. The following examples highlight some of the genetic studies in this population and the need for further study of the genetic and environmental basis for the disproportionate rates of dyslipidemia. The differences in MAF have been highlighted by the 1000 Genomes Project, and have informed databases such as the dbSNP database of National Center for Biotechnology Information (NCBI) (https://www.ncbi.nlm.nih.gov/snp) [60]. One such example of major differences in MAF and CVD risk is rs1800588 (*LIPC*). This SNP has a global MAF of 0.39, but is as high as 0.50 in Mexican populations (1000 Genomes). The rs1800588 genotype has been associated with TG concentrations among Mexican adults [61]. The current MAF and risk allele definitions for Mexican populations that come from the 1000 Genomes Project is from 107 individuals living in Los Angeles, United States (US). This is not a large enough sample to generalize for the entire Mexican population in both the US and Mexico. Our group has published on a larger cohort study of almost 1000 individuals from Mexico, and have detected differences in minor and risk alleles for SNPs in the *FADS* cluster [53]. Furthermore, a recent GWAS conducted in Mexicans identified novel genetic loci to be associated with TG concentrations [62]. These variants had not been identified in previous studies, thus emphasizing the need for further investigation into the genetic effects of dyslipidemia in this understudied, at-risk group. The lack of diversity in genomic research is limiting the implementation of precision medicine and nutrition recommendations for people of diverse ethnicities [63].

The lack of reproducibility among genetic association studies is particularly problematic for diverse populations. These ethnic subgroups, such as Hispanic and African-Americans, have some of the highest prevalence of dyslipidemia and other chronic, non-communicable diseases in the US [64]. The results from genetic association studies conducted in populations of European ancestry may not translate to these diverse populations, delaying the benefit that these individuals might receive as medicine moves toward the direction of personalized, genotype-based recommendations.

## 4. Dietary Contributions to Dyslipidemia

Dietary intake has a crucial role in affecting metabolic health and disease risk. Dietary components that have been previously implicated in increasing blood lipid concentrations include alcohol, carbohydrates, and dietary fat. The role of alcohol [65] and carbohydrates [66] have been previously reviewed; therefore, the current review will focus on dietary fat. Currently, agencies such as the American Heart Association (AHA), the Department of Agriculture, and the Department of Health and Human Services to recommend the limiting of total and saturated fat (SFA) [2,67]. However, the recommendation of a low-fat diet for heart health was challenged when researchers from the Seven Countries Study observed a low prevalence of CVD in the Mediterranean region, despite the consumption of a diet containing a moderate amount of total fat, coming from olive oil and cold-water fish [68,69]. This led to several seminal clinical trials to explore the effects of the Mediterranean diet on CVD risk and mortality, such as the Lyon Heart Study and PREDIMED [70,71]. The promising results of these trials have led to further scientific exploration of differential effects of various types of dietary fatty acids in ameliorating or exacerbating CVD risk. The replacement of saturated for unsaturated fat in the diet has been evidenced to be lipid-lowering and protective against CVD [72,73].

Unsaturated fats, those present in high amounts in the Mediterranean diet described above, are classified into monounsaturated (MUFA) and polyunsaturated (PUFA) fatty acids. MUFAs are present in foods such as avocados, almonds and other nuts, and vegetable oils. MUFAs are cardioprotective in that they do not raise blood lipid concentrations, and are less susceptible to oxidation than are PUFAs, due to their lower degree of unsaturation [74]. MUFAs are also more effective than carbohydrates in reducing blood lipid concentrations when replaced for SFA in the diet [75]. The majority of studies examining MUFA intake and blood lipids have been conducted concurrently with consumption of a Mediterranean diet, which has been associated with lower TG concentrations in meta-analyses [76,77]. However, as there are additional dietary components present in a Mediterranean diet, such as fiber, micronutrients, phytochemicals, and PUFAs, it is difficult to elucidate the specific effects of MUFAs alone on blood lipids. The effects of PUFA intake, present in cold-water fish, walnuts, and corn oil, on blood lipids and CVD risk have been studied extensively [78]. Intake of n-3 and n-6 PUFAs have both been associated with decreased CVD risk, especially the n-3 series, as they have been evidenced to have anti-arrhythmic and potent TG-lowering effects [79]. N-3 PUFA supplementation is currently recommended by the AHA to prevent recurrence of myocardial infarction [80].

The mechanisms by which unsaturated fatty acids affect blood lipid profiles have been previously summarized [70,72,74,81]. In brief, PUFA can serve to upregulate mRNA and thus protein levels of LDL receptors, resulting in increased lipoprotein uptake to the liver [82]. PUFA also downregulate fatty acid synthase, a key step in *de novo* lipogenesis, and very-low density lipoprotein (VLDL) secretion from the liver [83]. MUFA has been associated with decreased apolipoprotein C-III, which is an activator of LPL [84]. Clinical studies have also shown the effects of MUFA intake on decreasing apoB-100 production, the primary apolipoprotein present on circulating VLDL [81]. 

The lipid and lipoprotein response to intake of these various fatty acids may not be consistent among different populations studied. The reasons for this variability can be due to age, sex, disease state, differences at the genetic level, or any combination of these factors. The role of individual genetic variation in determining differential phenotypes has become more clearly understood, as advances in genetic technology and large cohort studies have identified significant associations between dietary intake and genetic variants to produce differences in disease risk [85]. The field of nutrigenomics refers broadly to the study of the interactions between dietary intake and the genome [86]. These interactions can result in epigenetic modification of genes, transcriptional regulation, or alterations in protein functionality. A subset of nutrigenomics is nutrigenetics, which specifically examines the effect of individual genetic variants (i.e., SNPs) and dietary intake on phenotypic expression. The exploration of these interactions can direct the creation of personalized recommendations for consumption of certain dietary fatty acids for the maintenance of normal lipid profiles and achievement of a healthy weight.

## 5. Nutrient-Gene Interactions and Dyslipidemia

Nutrigenetics, the science of the effect of genetic variation on response to dietary intake, bridges the gap between biological (genetic) and behavioral (diet) factors contributing to complex diseases, and can offer explanation as to why researchers may observe differential effects among individuals fed identical diets. Knowledge gained from this field is promising, as it can lead to explanation of response variability in clinical trials with diverse populations, better identification of non-responders to various diets, and the development of personalized dietary strategies [87].

As dyslipidemia is the result of a combination of genetic and environmental factors, it is logical that these two elements be examined in conjunction with one another. As many genetic loci associated with dyslipidemia have already been identified, the next step is to identify gene-environment interactions that may exacerbate or ameliorate the effect of genetic variation on disease risk. The integration of environmental exposures, especially dietary intake, may be able to add to the understanding of the complex etiologies associated with dyslipidemia. Furthermore, a targeted approach through the selection of physiologically relevant genes involved in pathways of lipoprotein metabolism and atherosclerosis will yield important discovery into the biology behind how these proteins metabolize nutrients in the presence of genetic mutations [14].

Previous research conducted to further classify the effects of common variants and dietary fat intake on lipid profiles has provided initial evidence of the need for deeper understanding in this field. Current literature on gene-diet interactions of physiologically relevant genes and dietary fat intake is presented in Table 2. A classic example, from Ordovas et al. examined the interactive effects of a polymorphism in the promotor region (rs1800588) of the *LIPC* gene and total fat intake [88]. This polymorphism has been associated with decreased activity of hepatic lipase. Mutations in this enzyme can result in elevated TG. Results indicated that rs1800588 genotype was significantly associated with HDL concentrations, and this association was strengthened when dietary intake was also considered. The interaction between presence of the risk allele and fat intake greater than 30% of total calories was associated with increased HDL, suggesting that these individuals may benefit from a high fat diet, specifically one high in MUFAs [89]. A diet high in total fat, defined in a study by Sanchez-Moreno et al. as consumption greater than 98 grams per day (the study median), did not associate with significantly higher TG concentrations by *APOA5* (rs662799) genotype, indicating there was no disadvantage to this mutation in individuals’ ability to metabolize a high-fat diet [90]. These studies indicate that a high-fat diet may be beneficial in maintaining desired blood lipid concentrations for individuals possessing the minor alleles of common variants.

The nutrigenetic interactions between n-3 PUFA intake and common variants in genes related to atherosclerosis are summarized by Merched and Chan [91]. PUFA intake has been evidenced to interact with a polymorphism in *PPARA,* a transcriptional regulator of lipid metabolism, to associate with lower TG concentrations among those consuming high PUFA intake [92]. Several variants in *APOA5*, coding for apolipoprotein AV, have been evidence to interacte with PUFA intake to associate with elevated TG concentrations in individuals possessing the risk allele [93]. However, the *APOA5* gene has 14 known SNPs listed on the NCBI database alone, so it is quite possible that these variants may interact with one another, or other genetic or non-genetic factors, to affect response to dietary intervention. 

As we recognize the need to translate basic science into clinical applications, individual genetic variants have been studied as predictors of the lipid response to dietary interventions among individuals with obesity [94,95]. Several studies have specifically examined the role of physiologically relevant variants on changes in lipid profiles, with promising results. Zhang et al., studied the role of the rs964184 variant (*APOA5*) on modifying changes in TC, HDL, and LDL after the POUNDS LOST (Preventing Overweight Using Novel Dietary Strategies) weight loss trial, in which participants were randomized to follow one of four diets of varying macronutrient composition [96]. However, the POUNDS LOST dietary conditions varied in total fat content, and the researchers did not account for the dietary fat composition (SFA, MUFA, PUFA) of the diets. The type and amount of dietary fat intake is evidenced to interact with genetic variants to influence blood lipid profiles [97,98,99,100].

The gene-diet interactions of cholesterol metabolism were reviewed by Abdullah et al., in 2015 [101]. These researchers focused their review on the outcomes of TC, LDL, and HDL concentrations and any dietary exposure. This review provides strong evidence for the role of variants in over 20 genes involved in cholesterol metabolism. Nuno et al. recently reviewed the literature on variants in genes involved in lipid metabolism and dietary intake on CVD risk [102]. Many of the studies reported in this review describe significant associations with variants in several of the genes mentioned, blood lipids, and dietary intake of carbohydrates and various fatty acids. One notable limitation in these reviews is the limited research on dietary patterns, rather than consideration of macronutrient intake alone. As nutrients are not consumed in isolation, the study of the interacting effects of different dietary components, as in a Mediterranean diet, with individual genetic variation on disease risk is a promising direction for future study. Additionally, as fatty acids are heterogeneous, there must be more research on the interacting effects between different classes of fatty acids (medium- to long-chain, degree of saturation, etc.) and the foods they are present in and genetic variation. A relevant example of this is dairy fat. Dairy consumption has been associated with protective benefits against T2DM, obesity, and other cardiometabolic biomarkers [103,104]. A recent longitudinal study of over 2000 adults concluded that circulating levels of the fatty acids present in dairy products (pentadecanoic, heptadecanoic, and trans-palmitoleic) were not associated with mortality, but were associated with lower risk of CVD mortality [105]. More research into the effects of various foods high in SFA on risk factors for metabolic diseases are warranted, as these fatty acids are not consumed in isolation, and even foods high in SFA contain some degree of unsaturated fat.

These nutrient-gene interactions only represent a portion of the functional variants that have been associated with blood lipids in GWAS. Nevertheless, additional research is necessary, especially in diverse or minority populations across the lifecycle, to further elucidate the mechanisms by which diet can interact with genetic variation. The ultimate goal of this field of research is the translation of these discoveries into personalized nutrition recommendations to treat and prevent disease [106].

## 6. Conclusions

The genetic, dietary, and nutrigenetic components described here highlight the strong relationships between biological and behavioral risk factors for dyslipidemia. The exploration of physiologically relevant variants and their interactions with dietary lipids is especially pertinent to the development of personalized dietary recommendations for management of dyslipidemia. As obesity, a major risk factor for the development of dyslipidemia, continues to increase in prevalence worldwide, effective strategies to achieve a healthy weight and manage lipid profiles are needed. Due to genetic variation, among other factors, not all individuals will respond uniformly to these strategies. Thus, the identification of factors that explain this variability in response will provide researchers and clinicians with the information to apply targeted treatment approaches to maximize benefits against dyslipidemia. Understanding the biological reasons behind why an individual may not respond is a key research priority to be addressed, as this can lead to implementation of individualized nutrition recommendations that can be implemented to prevent and treat dyslipidemia [107].

## Figures and Tables

**Table 1:** Monogenic disorders affect blood lipid concentrations (not an extensive list).

Phenotype	Disorder	Gene Affected	Prevalence
High LDL	Hyperlipoproteinemia Type 2A	*LDLR*	0.2%
	Autosomal Dominant Hypercholesterolemia	*PCSK9, APOE*	0.5%
Low HDL	Tangier Disease	*ABCA1*	<100 cases reported worldwide
	Familial LCAT deficiency	*LCAT*	70 reported cases
High TG	Familial Chylomicronemia	*LPL, APOC2*	<0.0001
	Severe Hypertriglyceridemia	*APOA5, LMF1*	<0.5%

Table adapted from Dron and Hegele [15]. LDL, low-density lipoprotein cholesterol; HDL, high-density lipoprotein cholesterol; TG, triglycerides; LDL-R, LDL receptor; PCSK9, proprotein convertase subtilisin/kexin type 9; APOE, apolipoprotein E; ABCA1, adenosine triphosphate (ATP) binding cassette subfamily A member 1; LCAT, lecithin-cholesterol acyltransferase; LPL, lipoprotein lipase; APOC2, apolipoprotein C2; APOA5, apolipoprotein A5; LMF1, lipase maturation factor 1.

**Table 2:** Summary of gene-diet interactions between physiologically relevant variables of lipid and lipoprotein metabolism and dietary fat intake associated with blood lipids.

Gene	Locus	Protein Function	Previous Nutrient-Gene Interaction with Blood Lipids	SNP	Function of Variant	Risk Allele	MAF Global
Reverse Cholesterol Transport Pathway							
*CETP*	16q13	Facilitates the exchange of cholesterol esters for TG between lipoproteins in circulation	Total fat and TG [98]; total fat and TG [108]	rs5882	Missense variant	G	0.37
*ABCA1*	9q31.1	HDL-C bound protein that transports intracellular cholesterol onto HDL-C	Total fat and HDL [109]	rs9282541	Missense variant	T	0.01
			SFA and TG [108]	rs2230806	Missense variant	T	0.32
*LIPC*	15q21.3	Hepatic triglyceride lipase, also involved in lipoprotein uptake	SFA and HDL, TG [98]; total fat and HDL [110]	rs1800588	Intron variant in promotor region, associated with lowered LIPC activity	T	0.29
*APOA1*	11q23.3	Predominant apolipoprotein on HDL; activator of LCAT	SFA, total fat, and TC [108]	rs670	Upstream intronic variant	T	0.18
			Total fat and HDL [108]	rs5070	Intron variant	G	0.44
Cellular Lipid Uptake Pathway							
*APOE*	19q13.32	Present on TG-rich lipoproteins (chylomicrons, VLDL)	Total fat, SFA, and HDL [98]	rs405509	Upstream variant in promoter region	T	0.47
*CD36*	7q21.11	Scavenger receptor, binds to oxidized LDL and LCFA.	Oily fish (*n*-3 PUFA) and HDL [40]	rs6969989	Intron variant	G	0.33
*LPL*	8p21.3	Hydrolyzes TG to allow fatty acids from lipoproteins into circulation	Total fat and HDL [33,110]	rs328	Nonsense variant	G	0.10
Lipid/Lipoprotein Formation Pathway							
*APOA5*	11q23.3	Present on HDL particles, stimulates LPL, major determinant of plasma TG concentrations	Total fat and TC, LDL, HDL [96];	rs964184	3’ untranslated region (UTR) variant	G	0.22
			Total fat and TG [90]	rs662799	Upstream variant in promoter region	G	0.16
*FADS* Complex	11q12-13.1	Desaturation of long-chain fatty acids	*n*-3, *n*-6 PUFAs and HDL [111]; alpha-linolenic acid and non-HDL cholesterol [112]	rs174546	3’ UTR variant	T	0.28
*MLXIPL*	7q11.23	Activates carbohydrate-responsive element binding protein and promotes hepatic TG synthesis	Mediterranean diet and TG [113]	rs3812316	Missense variant	G	0.11
*PPARA*	22q13.31	Nuclear receptor in liver, ligand for PUFAs	*n*-3 PUFA and TC, LDL	rs6008259	Non-coding transcript variant	A	0.32
			*n*-6 PUFA and TC, LDL [114]	rs3892755	Non-coding transcript variant	A	0.09
